# Transforming Growth Factor-Beta and Matrix Metalloproteinases: Functional Interactions in Tumor Stroma-Infiltrating Myeloid Cells

**DOI:** 10.1155/2014/521754

**Published:** 2014-01-21

**Authors:** Jelena Krstic, Juan F. Santibanez

**Affiliations:** Laboratory for Experimental Hematology and Stem Cells, Institute for Medical Research, University of Belgrade, Dr Subotića 4, 11129 Belgrade, Serbia

## Abstract

Transforming growth factor-beta (TGF-**β**) is a pleiotropic factor with several different roles in health and disease. In tumorigenesis, it may act as a protumorigenic factor and have a profound impact on the regulation of the immune system response. Matrix metalloproteinases (MMPs) are a family that comprises more than 25 members, which have recently been proposed as important regulators acting in tumor stroma by regulating the response of noncellular and cellular microenvironment. Tumor stroma consists of several types of resident cells and infiltrating cells derived from bone marrow, which together play crucial roles in the promotion of tumor growth and metastasis. In cancer cells, TGF-**β** regulates MMPs expression, while MMPs, produced by either cancer cells or residents' stroma cells, activate latent TGF-**β** in the extracellular matrix, together facilitating the enhancement of tumor progression. In this review we will focus on the compartment of myeloid stroma cells, such as tumor-associated macrophages, neutrophils, and dendritic and mast cells, which are potently regulated by TGF-**β** and produce large amounts of MMPs. Their interplay and mutual implications in the generation of pro-tumorigenic cancer microenvironment will be analyzed.

## 1. Introduction

Transforming growth factor-beta (TGF-*β*) is a pleiotropic factor with several different roles in health and disease [1]. In cancer, TGF-*β* plays a paradoxical role, since it represses epithelial tumor development in the early steps of tumorigenesis, while in advanced stages it can stimulate tumor progression [2, 3]. In epithelial cells, TGF-*β* has antiproliferative and apoptotic roles which enable it to reverse local mitogenic stimulation in the pretumoral stage in the epithelium [4]. During the advance of tumorigenesis, carcinoma cells acquire resistance to the proliferative inhibition and apoptosis induced by TGF-*β*. Several mechanisms have been described to explain the changes in the response of tumor cells to TGF-*β*1, including mutations in the machinery of TGF-*β* signaling, as described below. Interestingly, the pro-tumoral role of TGF-*β* can be achieved either by acting directly on carcinoma cells or by modulating the crosstalk between cancer cells and noncancer cells in the tumor stroma [5]. TGF-*β* is produced by carcinoma cells as well as by the varied tumor stroma-associated cell populations, such as mesenchymal cells and immune cells (macrophages, neutrophils, mast cells, myeloid precursors, and T cells, among others). Therefore, TGF-*β* is accumulated in tumor stroma because of the oncogenic activation of tumor cells and/or as a consequence of the infiltration of TGF-*β*-secreting inflammatory cells, finally resulting in the enhancement of tumor malignance [6].

During the advance of tumor, carcinoma cells acquire the capacity to migrate and invade surrounding tissues and colonize different organs during metastasis. Matrix metalloproteases (MMPs) are a group of mainly extracellular matrix (ECM) proteolytic enzymes which enable cells to migrate and invade [7, 8]. Many of MMPs are tightly transcriptionally regulated in normal development but are deregulated in cancer [4]; therefore, their activity and expression are related to the worsening in the development of cancer. The expression of MMPs within tumor stroma is diverse; members of MMPs can be produced by cancer cells, but the major source of these enzymes is the stromal cells of the tumor, including infiltrating immune cells. Different sets of MMPs are produced by different types of stroma-associated cells and collectively collaborate to alter the milieu inside and around the tumor [9, 10]. TGF-*β* modulates MMPs expression in both cancer cells and tumor stroma-associated cells, while in the tumor microenvironment MMPs activate the latent secreted TGF-*β*, producing a harmful cycle which contributes to the worsening of tumor malignance. Here we review the specific roles and the functional interplay of TGF-*β* and MMPs in tumor stroma-associated myeloid linage of immune cells. The heterotypic reciprocal interaction among TGF-*β*, MMPs, and immune cells can function as the promoter of the events which enhance tumor progression and metastasis suggesting combined therapies for cancer treatment.

## 2. Transforming Growth Factor Beta

**Figure 1:**
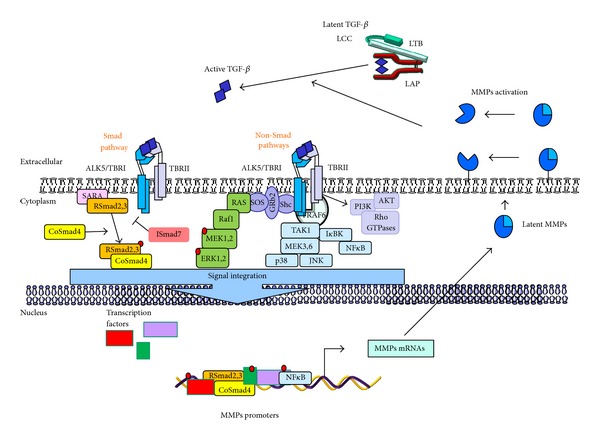
TGF-*β* signaling and MMPs interplay. Active TGF-*β* binds to its cell-surface type II receptor (TBRII), inducing the activation of TGF-*β* type I receptor (ALK5 or TBRI) and forming a heterotetrameric complex. Then two sets of signaling pathways can be stimulated: the Smad pathway, where ALK5 phosphorylates Smad2,3 and promotes the release of Smads from the complex with SARA from the inner face of the plasma membrane (phosphorylated Smad2,3 interact with co-Smad4, forming a heteromeric complex to be translocated into the cell nucleus) and non-Smad pathways, where active TGF-*β*-receptor complex interacts with ubiquitin ligase tumor necrosis factor receptor-associated factor 6 (TRAF6) which in turn recruits TGF-*β* activated kinase 1 (TAK1) to activate p38, JNK, or NF*κ*B pathway. On the other hand, TGF-*β* binding provokes the phosphorylation of ALK5 at tyrosine residues which enable the formation of Shc-Grb2/SoS complex to activate Ras-Raf1-MEK1,2-ERK1,2 signaling. Finally, receptor activated complexes can activate PI3K, provoking the activation of AKT and the small Rho GTPases. The activation of both Smad and non-Smad signaling pathways in turn initiate transcriptional or nontranscriptional activity to regulate MMPs expression, thus incrementing the protein levels in tumor microenvironment. When membrane bound MMPs or soluble MMPs are expressed, they may promote the activation of latent TGF-*β* by proteolytic cleavage within the N-terminal region of the latency-associated peptide (LAP) or the large latent complex (LLC).

Mammals express three genetically distinct isoforms of TGF-*β* (TGF-*β*1, TGF-2, and TGF-3) with high homology and their human genes are located on chromosomes 19q13, 1q41, and 14q24, respectively (revised in [1]). TGF-*β* initiates signaling by binding to cell-surface serine/threonine kinase receptors types I and II (TBRI and TBRII, resp.), which form a heteromeric complex in the presence of the dimerized ligand (Figure 1). Binding of TGF-*β* to TBRII leads to the phosphorylation of TBRI, thus activating its kinase domain [11]. When the receptor complex is activated, it phosphorylates and stimulates the cytoplasmatic mediators, Smad2 and Smad3 [12]. The phosphorylation of Smad2,3 releases them from the inner face, where they are specifically retained by Smad anchor for receptor activation (SARA). Further on, Smad2,3 form a heterotrimeric complex with the common Smad4, which is then translocated into the nucleus where, in collaboration with other transcription factors, it binds and regulates promoters of different target genes [1, 12]. TGF-*β*1 signaling can be regulated by the expression of other components of Smads, the inhibitory Smad proteins (I-Smads: Smad6 and Smad7). TGF-*β* regulates the expression of I-Smads, which establish a negative feedback loop to control TGF-*β* signaling. Essentially, Smad7 antagonizes TGF-*β* by interacting with TBRI and leading to its degradation [13]. In addition to Smad signaling, TGF-*β*1 may activate other intracellular signaling pathways, called non-Smad pathways, such as mitogen-activated protein kinases (MAPK): ERK1,2, JNK and p38; PI3K (phosphoinositide 3-kinase)/AKT1,2 and mTOR, known as cell survival mediators; NF-*κ*B (nuclear factor *κ*B), Cyclooxygenase-2, and prostaglandins; and the small GTPase proteins Ras, Rho family (Rho, Rac1 and Cdc42), among others [14, 15].

## 3. The Role of TGF-***β*** in Cancer

As already mentioned, TGF-*β* can act either as a tumor suppressor or as a tumor promoter. Suppression of tumor cell growth by TGF-*β* depends on its ability to upregulate the cyclin kinase inhibitors which inhibit cell proliferation. However, as the premalignant lesions progress, they become refractory to growth inhibition and begin to produce large amounts of TGF-*β*. Many malignant tumors have mutated or downregulated TBRII or other abnormalities in the TGF-*β* signaling pathways [2, 3]. The importance of TGF-*β* signaling in human cancers is evident from the frequent alterations of TGF-*β* signaling components in hereditary human cancers and sporadic cancers [16]: for example, the autosomal dominant familial juvenile polyposis syndrome (JPS) is the most common of the hamartomatous syndromes which occurs with an incidence of about one per 100.000 births [16–18] and germline mutations in different members of the TGF-*β* superfamily have been described in JPS patients. Around 15–20% of patients have Smad4 mutations, predominantly in MH2 domain [19, 20]. In the autosomal dominant disorder hereditary nonpolyposis colorectal cancer (HNPCC), the most common hereditary predisposition for the development of colorectal cancer, TBRII gene contains a 10-base pair polyadenine repeat microsatellite sequence, and up to 80% of colon cancer patients with HNPCC present this mutated form of TBRII [21]. In sporadic cancer, the specific response to TGF-*β* during tumor progression will depend on the stage of carcinogenesis and the responsiveness of tumor cells, and can be attributed to both independent and interrelated factors including changes in: (1) TGF-*β* expression; (2) receptor expression; (3) availability of downstream signaling components; (4) evasion of the immune response; (5) stimulation of inflammation; (6) presence of local and systemic factors (autocrine, endocrine, paracrine, juxtacrine, or matricrine interactions); and (7) the recruitment of cell types that lead to advanced tumor growth or promote angiogenesis [2].

Several tumors express high levels of TGF-*β*s, which correlate with tumor progression and clinical prognosis, making the concerning TGF-*β* family members diagnostic, prognostic, or predictive markers. Hence, increased serum levels of TGF-*β*1 have been considered as a prognostic marker of advanced disease and poor prognosis in multiple cancer types such as gastric carcinoma, colorectal cancer, bladder carcinoma, prostate cancer, breast cancer, lung cancer, esophageal adenocarcinoma, and melanoma. However, TGF-*β* levels are not yet used as tumor markers in clinical routine [18].

The expression of TGF-*β* receptors within tumor cells can also be used as prognostic markers. For example, reduced ALK-5/TBRI expression has been described in colon cancer patients, and two polymorphisms in TBRI (TBRI*6A and Int7G24A) have been identified in patients with breast cancer [16]. Meanwhile, low expression levels of TBRII have been observed in chronic myeloid leukemia and colon cancer patients, and mutations in TBRII are frequently found in colon cancer, gastric cancer, ovarian cancer, and nonsmall cell lung carcinoma [22]. In a majority of human pancreatic cancers mutations in TBRI and TBRII that alter protein and/or mRNA expression levels have been identified [16, 23]. In addition, low levels of TBRI/ALK5 and TBRII expression in breast cancer have also been associated with epigenetic silencing [24]. Conversely, some studies suggest that ALK5/TBRI and TBRII expression is increased in advanced human malignant glioma tissues compared to nontumorous gliosis. In addition, microsatellite instability which carried TBRII genetic mutations in a particular repeat region of the TBRII gene has been reported [25, 26]. Finally, TGF-*β* signaling promotes epithelial to mesenchymal transition, a characteristic of invasive and metastatic cells, leading to increased metastases in human cancer, as well as in animal cancer models [3].

## 4. Matrix Metalloproteinases

The notion that the remodeling of the ECM in tumor plays a strategic role in the increment of cancer cell malignance is becoming increasingly accepted. Therefore, ECM degrading proteases, such as MMPs, are gaining more attention as mediators of the alterations in tumor stroma, which can support cancer progression. In addition to the remodeling of ECM, MMPs exert different roles which are involved in the promotion or inhibition of tumor progression. MMPs can contribute to tumor cell proliferation by altering the bioavailability of growth factors, such as insulin growth factors (IGFs) and the epidermal growth factor receptor (EGFR) ligands. Both apoptotic and antiapoptotic actions of MMPs are also known; for example, the antiapoptotic signals are transduced to cancer cells by MMP-7's ability to cleave Fas ligand, a transmembrane stimulator of the death receptor Fas, from the cell surface. On the other hand, MMPs can also promote apoptosis, probably by modulating the ECM composition, for example, by cleaving laminin, which influences integrin signaling. Some MMPs, like MMP-2, MMP-9, and MMP-14, can act both as positive and negative regulators of angiogenesis in tumor vasculature. Moreover, MMPs can promote EMT by proteolytic activation of TGF-*β*, and the same activation can be involved in the suppression of T-lymphocyte reaction against cancer cell proliferation (revised in [10, 27]).

Matrix metalloproteinases family consists of more than 20 zinc-dependent endopeptidase enzymes, sharing a similar structure, with the ability to degrade almost all components of the ECM. MMPs facilitate tissue remodeling and normal and pathological cell migration, functions that allow malignant cells in cancer to invade and move through the matrix barrier. Based on their domain structure and substrate preference, MMPs are traditionally grouped into (1) collagenases, including MMP-1, -8, -13; (2) stromelysins, MMP-3 and MMP-10; (3) gelatinases, MMP-2 and MMP-9; (4) matrilysins, MMP-7 and MMP-26; (5) membrane-type MMPs (MT-MMPs); and others (Table 1) [28].

All MMPs are synthesized as zymogens or pro-MMPs and, with the exception of the membrane bound MT-MMPs, are secreted and can be activated by a proteolytic cleavage mechanism [28]. The proteolytic activity of MMPs is mainly regulated by tissue inhibitors of MMPs (TIMPs). Four TIMPs (TIMP-1, TIMP-2, TIMP-3, and TIMP-4) have been described and they can inhibit all active MMPs, however, not with the same efficacy [29].

It has recently been postulated that some MMPs have a protective role in cancer. Mainly, the tumor suppressor role of these MMPs is related to their ability to degrade plasminogen, collagen XVIII, and collagen IV to produce natural angiogenic inhibitors, such as angiostatin, endostatin, and tumstatin [4]. MMP-8 expression can be used as a good prognostic marker in breast cancer, while MMP-3, MMP-11, and MMP-19 have been found to play dual roles in cancer, and they may exert protumorigenic or suppressor roles depending on the tumor context [30].

## 5. MMPs Activate TGF-***β*** in the Extracellular Matrix Compartment

TGF-*β* and MMPs may function in a bidirectional regulatory loop associated with cancer development, TGF-*β* needs to be proteolytically activated by MMPs in order to exert its cellular functions, whereas activated TGF-*β* in tumors modulates the balance of ECM remodeling by regulating the expression of MMPs and their tissue inhibitors TIMPs [10, 31]. TGF-*β* is translated as a homodimeric precursor, a 75 kd protein, which consists of the signaling peptide, latency-associated peptide (LAP), and the mature TGF-*β*. Intracellularly, the precursor is cleaved by the furin-type convertase producing the small latent complex (SCL), where the 25 kd dimeric TGF-*β* remains attached to LAP. Next, SCL and the latent TGF-*β* binding protein (LTBP) form the large latent complex (LCC), which after secretion remains covalently associated with the ECM. In the ECM, the activation of TGF-*β*1 involves the proteolytic cleavage of LAP by soluble MMP-9 or MMP-9 bound to CD44 in the cell-surface, MMP-2, MMP-13 or MMP-14 [32, 33]. Given that cancer cells frequently develop resistance to the TGF-*β* suppressive tumor effects, it is suggested that the activation of TGF-*β* by MMPs can have profound tumor-promoting effects by selectively driving stroma-mediated invasion and metastasis of the tumor [10].

## 6. Regulation of MMPs Expression by TGF-***β***

TGF-*β* is able to stimulate several MMPs in cancer cells. This effect on MMPs can be due to its capacity to activate a plethora of signal transduction pathways and different transcription factors other than Smads, showing the complexity in the capacity of TGF-*β* to regulate MMP expression in cancer cells. Smads and several other response elements in MMP promoters by which TGF-*β* may control the MMPs expression at transcriptional levels have been described.

At least two different regulatory domains have been described: (1) TGF-*β* inhibitory element, TIE, (2) and the Smad binding element (SBE) [1, 34–36]. Since MMP-1, MMP-7, MMP-9, MMP-13, and MM-P14 contain TIE binding sites in their promoters, it is postulated that the expression of these MMPs may be modulated by TGF-*β* [37, 38]. It has been demonstrated that TGF-*β* negatively regulates the transcription of MMP-1 and MMP-7 [39–41]. Conversely, molecular analysis revealed that the consensus TIE in the promoters of MMP-9, MMP-13, and MMP-14 was not required for their induction by TGF-*β* [42–44]. In addition, Smads, by interacting with the members of the AP1 family, can alter MMPs expression [45–47]. TGF-*β* regulates MMP-13 gene expression partly via the AP1 site and partly through interactions of Smad3 with JunB and Runx-2 [48]. TGF-*β* also directly activates other transcription factors implicated in the regulation of MMPs expression; it induces cell signaling that culminates in the transactivation of AP1, PEA3, NF*κ*B, SP1, and MEF-2 transcription factors to enhance MMP promoters transactivity [45, 49–52].

TGF-*β* activates a plethoric set of intracellular signaling pathways that may explain its wide role in cancer, as well as its profound impact in the regulation of MMPs (Figure 1). For example, TGF-*β* can induce the expression of MMP-2 by activating TAK1-p38 MAPK in breast epithelial cells [52, 53], while it enhances SW1990 invasiveness by stimulating MMP-2 expression through the activation of Rac1/ROS/NF*κ*B [54]. Meanwhile, MMP-9 has been shown to be upregulated by TGF-*β* through the activation of ERK1,2, Rac1-ROS-NF*κ*B, and TAK1-NF*κ*B in transformed keratinocytes, breast, and hepatocellular carcinoma cells [55–59].

## 7. The Roles of TGF-***β*** and MMPs in Tumor Stroma

Tumors present a highly dynamic integral entity; therefore it is becoming more apparent that the study of the molecular processes in tumor biology requires paying attention to all the components of the tumor. For many decades studies have been developed in the point of view of transformed cancer cells, which have been the main targets for oncotherapies. Transformed cells show the ability to interact with their microenvironment, and these mutual interactions are what may drive the tumor progression.

In solid cancers, the tumor stroma, tumor microenvironment, or host tissue stroma can be defined as the nonepithelial compartment surrounding the tumor cells [5, 60]. The tumor stroma is a complex structure consisting of the basement membrane and ECM supportive proteins, including extracellular molecules such as cytokines and growth factor; fibroblasts and cancer associated fibroblast (CAFs); tumor vasculature to supply nutrients and oxygen (lymphatic and blood vessels); and immune cells from the lymphoid (T lymphocytes) and myeloid linages (tumor associated monocytes/macrophages, neutrophils, dendritic cells, and myeloid precursor cells) [61–63]. Tumor stroma is a dynamic structure whose interactions with the epithelial cancer cells result in reciprocal influence. Interestingly, stroma cells may generate a tolerant or inductive microenvironment for tumor progression; for example, the interaction between tumor cells and stroma cells, such as CAFs and myofibroblast, triggers the secretion of growth factors and cytokines for the stimulation of tumor angiogenesis and may induce profound ECM modifications, sustaining cancer initiation, progression, growth, survival, and finally the metastasis process [61, 62, 64].

**Figure 2:**
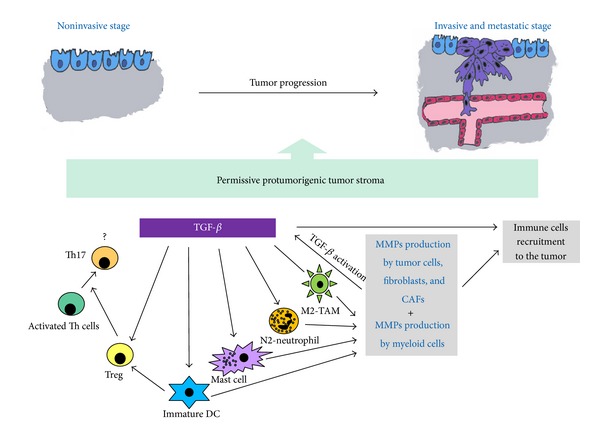
TGF-*β*-MMPs system and tumor stroma-infiltrating myeloid cells interplay during tumor progression. MMPs produced by resident tumor stroma cells, such as cancer associated fibroblast (CAFs) or pericytes, may activate latent TGF-*β*, and both can enhance the recruitment of immune cells, leukocytes, and myeloid cells to the tumor stroma. TGF-*β*, in turn, may regulate myeloid cells phenotype, to promote the protumorigenic M2 phenotype of tumor-associated macrophages (M2-TAMs) and the N2 phenotype of the tumor associated neutrophils (N2-TANs). At the same time, TGF-*β* regulates mast cells degranulation and stimulates the expression of IL-6. In addition, mast cells produce TGF-*β*, incrementing the level of the factor in tumor stroma. Meanwhile, TGF-*β* maintains dendritic cells (DC) in an immature stage, which in turn, can activate regulatory T cells (Tregs) to enhance immunosuppression. Stimulated Tregs in concert with mast cell produce IL-6 and induce the differentiation of Th17 cells which in turn may collaborate in the proinflammatory tumor response. Tumor stroma-infiltrating myeloid cells also produce large amounts of MMPs which can intensify TGF-*β* activation, and the TGF-*β*-MMPs-myeloid cells interplay to induce a tumor growth permissive stroma supporting tumor progression, thus strengthening the cancer cells invasion and metastasis.

Deregulation of TGF-*β* and MMPs expression and activation is frequently observed in tumor tissues and has been shown to contribute to tumor progression [2, 65]. As mentioned before, TGF-*β* acts as tumor suppressor in early stages of cancer development, while it promotes tumor progression and metastases in late stages. Interestingly, ablation of T*β*RII in stromal fibroblasts promotes carcinoma growth and invasion, indicating a regulatory loop of TGF-*β* signaling between epithelial cancer cells and the stromal compartment (revised in [2]). MMPs are mainly involved in the recycling of ECM as well as in releasing growth factors and cytokines from ECM. TGF-*β* is a potent inductor of the chemotaxis of immune cells within tumor, stimulating the migration of lymphocytes, monocytes, and neutrophils, among other cells [66]. In turn, these cells produce and secrete into the tumor stroma several MMPs [10, 65], which can release and activate latent ECM-associated TGF-*β* and also regulate immune cells function, together supporting cancer cells escape from immune surveillance (Figure 2) [27].

TGF-*β* and MMPs form an intricate network, regulated on many levels, which is why the perturbations in this network can contribute to the pathogenesis of malignant tumors. The contribution of TGF-*β* and MMPs expression and secretion in tumor is confined to all cellular components; therefore, we will next discuss the interplay between TGF-*β*, MMPs, and immune cells with the emphasis on monocytes/macrophages, neutrophils, and dendritic cells, which together contribute to tumor development.

## 8. TGF-***β*** and MMPs Functionality in Immune Cells within the Tumor Microenvironment

### 8.1. Inflammation Can Initiate and Promote Tumor Growth

In 1986, Dvorak [67, 68] described human tumors as “wounds that do not heal.” Intriguingly, the changes occurring in the tumor stroma during cancer progression resemble the process associated with chronic inflammation [68]. The primary role of the inflammatory response is to restrain and eliminate harmful body aggressors; such is the case of transformed cancer cells and the subsequent recruitment of antitumor immune cells to the cancer occurring tissue [68]. Beside the primary inflammatory response, chronic inflammation may be beneficial for the tumor development, since the changes in the tumor microenvironment can convert inflammation response to a tumor-supporting inflammatory process.

Several cancer types have been proven to be in correlation with previous chronic inflammatory conditions, manifested due to infection (e.g., *Helicobacter pillory* and gastric cancer), cryptogenic inflammatory states (e.g., chronic prostatitis and prostate cancer) or autoimmune diseases (e.g., inflammatory bowel disease and colon cancer), [69, 70]. However, tumors that are not potentially induced by inflammation are also characterized by the presence of the inflammatory component in their microenvironment [71].

### 8.2. Inflammatory Microenvironment in Tumor

The inflammatory microenvironment in the tumor includes the presence of host leukocytes both in the supporting stroma and among the tumor cells, with macrophages, dendritic cells, mast cells, neutrophils, and T cells being differentially distributed [63, 70, 71]. Besides the cellular component, this microenvironment is rich in inflammatory cytokines, growth factors, chemokines, and matrix degrading enzymes, which again induce the recruitment and the specific behavior of the cellular part.

## 9. TGF-***β*** and MMPs in Inflammation

TGF-*β* plays an important role in the control of inflammation. It may negatively regulate immune cell response by activating regulatory T cells (Tregs) and by inhibiting immune cells proliferation, while it can induce Th17 differentiation and enhance the secretion of proinflammatory cytokine IL-17 [66, 72]. One of the first studies implying TGF-*β* as a regulator of inflammation is coming from the characteristics found in TGF-*β* knockout mice, which died from multifocal inflammation and autoimmune disorders in internal organs, suggesting TGF-*β* as an immune suppressive factor [66]. Multiple types of immune cells can be regulated by TGF-*β* (revised in [72]). The following mechanisms are proposed: (1) suppression of the effector Th cell differentiation; (2) conversion of naive T cells into regulatory T cells; (3) inhibition of the proliferation of T cells and B cells; (4) inhibition of the effector cytokine production, such as IL-2, IFN-*γ*, and IL-4; and (5) suppression of macrophages, dendritic cells (DCs), and natural killer (NK) cells.

Increase in MMPs expression has been seen in almost every human tissue suffering inflammation (revised in [73]). Diverse inflammatory processes regulated by MMPs are dependent on multiple factors, such as MMPs expression, location, proteolytic activity, and substrate availability. MMPs may be involved in the degradation and remodeling of physical barriers and modulation of inflammatory mediators such as cytokines and chemokines, and by establishing chemokine gradients in inflamed tissues they can regulate the movement of immune cells to the affected tissues [10, 73]. Immune myeloid-derived cells, including macrophages, neutrophils, dendritic cells, and mast cells, produce and secrete MMPs within tumor stroma, which in turn exert and control multiple processes controlling tumor angiogenesis, cancer growth, and metastatic dissemination [10, 65].

## 10. Tumor Stroma-Infiltrating Myeloid Cells

Myeloid cell subpopulations, such as neutrophils, monocytes/macrophages, and dendritic cells, are key mediators of inflammatory and innate immune response (revised in [74]). Although these subpopulations share phagocytic activity, immunophenotype, origin, and cell turnover, they display different roles in inflammation, as neutrophils can amplify inflammation by releasing cytotoxic granules, whereas macrophages terminate inflammation and restore tissue integrity after removal of the inflammatory stimuli [74–76]. Myeloid cells also play important roles in adaptive immune responses during inflammation, since dendritic cells can activate antigen specific T cells, while neutrophils and macrophages suppress T-cell response, thus leading the concept of myeloid suppressor cells (MDSCs).

Tumors present an environment of unresolved inflammation initiated by malignant cells. During tumor development, myeloid cells accumulate massively within the tumor and regulate inflammatory and immune responses [74]. Further on, we will focus on the role of TGF-*β* and MMPs in myeloid-infiltrating tumor stroma.

### 10.1. Tumor-Associated Macrophages

The major components of the inflammatory lymphoreticular infiltrates of tumors are tumor-associated macrophages (TAMs) which have been implicated in tumor proliferation, progression, and invasion.

TAMs are initially mobilized from the bone marrow and recruited to the tumor site by specific tumor-derived stimuli and inflammatory CC chemokines (e.g., CCL2), while their recruitment and survival are sustained by cytokines present in the tumor microenvironment (e.g., CSF and VEGF-A) [70]. High TAM content in human epithelial tumors usually links to poor prognosis with few exceptions [77]. The critical function of TAMs has also been documented in the MMTV-PyMT transgenic mouse model of breast cancer. Crossing the PyMT breast cancer mice with the macrophage-deficient CSF1op/op mice suppressed tumor angiogenesis, tumor progression, and lung metastasis, whereas primary tumor growth remained largely unaffected [78].

Macrophages can exert dual functions in the context of tumors. Namely, TAMs can express M1 or M2 phenotype depending on the specific stimuli in their microenvironment. M1 macrophages activated by interferon and bacterial products can elicit tumor and tissue-destructive reactions by targeting cancer cells and the tumor vasculature and can therefore be acknowledged as “tumor destructing.” In contrast, in response to tumor derived or lymphocyte derived signals [79, 80], TAMs infiltrate the tumor tissue where they are set in an M2, alternative activation mode, promoting cell proliferation, angiogenesis, and tissue remodeling, thus being “tumor promoting.” M1 and M2 cells are extremes in a continuum of functional states [70, 81, 82] susceptible to change dependent on the factors present in their microenvironment.

TGF-*β* promotes recruitment of monocytes and it may promote monocyte to macrophage differentiation [2]. In the innate immune response, TGF-*β* promotes TAM polarization to an M2-versus-M1 phenotype, which further promotes TGF-*β* production and deepens immunosuppression [83, 84].

In response to cytokines such as TGF-*β*, IL-10, and macrophage colony-stimulating factor (M-CSF), TAMs can promote tumor proliferation and progression and stroma deposition, while also contributing to the remodeling and inhibition of adaptive immunity [85–87]. Several studies in human cancer have shown that TAMs accumulation is associated with increased angiogenesis and with the production of angiogenic factors such as VEGF and platelet-derived endothelial cell growth factor. It is believed that macrophages do not participate in angiogenesis unless activated and hypoxia is known to trigger a proangiogenic program in TAMs, as these cells accumulate in hypoxic regions of tumors. Furthermore, TAMs inhibit antitumor immunity by secreting TGF-*β*, leading to an increase in angiogenesis and the expression of growth factors involved in supporting tumor growth [63]. Many of the observed functional responses of macrophages to TGF-*β* may be attributed to the regulation of gene expression of inflammatory mediators. In macrophages, TGF-*β* has been shown to suppress the expression of MIP-1*α*, MIP-2, CXCL1, IL-1*β*, IL-8, GM-CSF, and IL-10 [2, 88].

Tumor invasion can be induced by macrophages through the production of enzymes and inhibitors that regulate the digestion of the ECM, as well as through the production of several MMPs including MMP-1, MMP-2, MMP-7, MMP-9, MMP-12 and MMP-14 [10]. For example, MMP-9 has complex effects beyond matrix degradation, including promotion of the angiogenesis switch and release of growth factors [69]. In a mouse model for cervical cancer, inhibition of MMP-9 in macrophages blocked the release of VEGF and thereby inhibited angiogenesis and tumor growth [89]. Although almost all of these MMPs have been implicated in the enhancement of tumor cell malignance by increasing angiogenesis and tumor invasiveness, MMP-12 has been implicated in the generation of angiostatin, and the mice deficient in MMP-12 showed an enhancement in the development of Lewis lung carcinoma metastases, suggesting TAM-produced MMP-12 as suppressive for the growth and malignance of lung cancer cells [90]. In addition, TGF-*β*1 inhibited cytokine-mediated induction of MMP-12 mRNA, protein, and enzymatic activity induced by cytokines [91]. TAMs also produce factors, such as PDGF, IL-6, uPA, and tissue-type plasminogen activator (t-PA) that may cause matrix degradation.

These data strongly suggest that macrophages play a role in both angiogenesis initiation in avascular areas and in the remodeling of the vasculature once formed to give coherent vascular flow [86]. TGF-*β*1 also increases the expression of fetal liver kinase-1 (Flk-1), a major VEGF receptor, and TGF-*β*1 and VEGF stimulate MMP-9 expression, respectively, thus increasing the invasiveness of cancer cells [92].

Finally, the specific deletion of TBRII in myeloid cells produces TAMs unresponsive to TGF-*β*, with downregulation in basal IL-6 secretion. Most importantly, TGF-*β* does not induce any VEGF response in these cells, and decreased MMP-9 and increased MMP-2 and iNOS expression were noted, along with the increase in antigen-antitumorigenic properties of TAMs [93].

### 10.2. Tumor-Associated Neutrophils

Neutrophils are the predominant circulating leukocyte population in humans, accounting for 50–70% of circulating leukocytes. They have been seen *in vivo* in close association with tumor cells and within tumor vasculature [94, 95]. Recently, neutrophils have emerged as new tumor-infiltrating myeloid cells, playing an important role in tumor growth and progression [96]. Similarly to macrophages, neutrophils may also have both protumoral and antitumoral roles [97].

Tumor-associated neutrophils (TANs) can stimulate tumor growth by stimulating angiogenesis through the production of proangiogenic factors, including VEGF, IL-8, and MMPs [98, 99]. Recent findings suggest the N1-N2 polarization model of TANs, similar to the M1-M2 model for TAMs [100]. This group suggested specific classification of TANs according to which these cells can have antitumorigenic (N1) phenotype versus protumorigenic (N2) phenotype.

Depletion of “protumorigenic” N2 neutrophils, therefore, inhibits tumor growth and reduces the level of immunosuppression in the tumor microenvironment, allowing increased activity of cytotoxic T lymphocytes [95, 101]. The antitumor activities of N1 type of TANs include expression of more immunoactivating cytokines and chemokines, lower levels of arginase, and more capability of killing tumor cells *in vitro*. Blockade of TGF-*β* favored the accumulation of N1 TANs, suggesting that TGF-*β* is a major proximal cytokine within tumors that defines the TAN phenotype and shifts differentiation toward the N2 protumorigenic phenotype [100]. Interestingly, preliminary data suggested that at least part of the neutrophil-attracting chemokines is derived from TAMs, suggesting a ‘‘ménage á trois” involving T cells, macrophages, and neutrophils [102]. Interestingly, TGF-*β* may inhibit neutrophils adhesion and endothelial cell transmigration *in vitro* and in different inflammatory disease state as well [95]. Another point of TGF-*β* regulation of the inhibition of suppressive roles of neutrophils is the capacity of TGF-*β* to decrease the ability of neutrophils to eliminate Fas ligand expressing cancer cells, one of the main functions of neutrophils in tumor microenvironment, thereby creating a stroma permissive for tumor progression [103].

Neutrophils have a role in the initiation of carcinogenesis, mainly by regulating the ECM turnover which can have profound effects on the tumor microenvironment. A key mediator secreted from neutrophils and involved in carcinogenesis is MMP-9. MMP-9 has been demonstrated to play key roles in tumorigenesis participating in the progression of cancer cells to more invasive and metastatic stages. Interestingly, MMP-9 secreted by neutrophils or by other cells in the tumor stroma may prevent apoptosis of cancer cells [95, 104].

Neutrophils' granules contain large amounts of MMP-9. Moreover, serine proteases released by these cells activate MT1-MMP, a membrane bound MMP that activates pro-MMP-2, leading to an increase in active MMP-2. On the other hand, MMPs can recruit neutrophils to sites of inflammation, which requires the extravascular migration of neutrophils through the extracellular matrix. Furthermore, TGF-*β*1 stimulates degranulation of adherent human neutrophils [105]. Enzymes released by the neutrophils within the tumor milieu can activate latent proteases and diminish cell-cell interactions, thereby permitting the dissociation of tumor cells from the main tumor mass. The embedded growth factors and chemoattractants released during ECM remodeling, such as basic fibroblast growth factor, could serve both as chemoattractants and growth factors for these tumor cells [106].

Conversely to the protumorigenic properties of neutrophils, several studies have reported the antitumorigenic roles for these cells. For example, during TGF-*β* inhibition, neutrophils can inhibit tumor growth by assuming a tumor-cytotoxic N1 phenotype, and depletion of these N1 TAN may either enhance tumor growth and/or decrease the antitumor effects of immunologic treatments [95, 100].

Neutrophils may also exert their antitumor activities by expressing and secreting MMP-8, which is mainly produced by neutrophils and has been postulated as a tumor suppressor [107]. The loss of MMP-8 increased skin susceptibility to chemical carcinogens in mice. Intriguingly, the absence of MMP-8 prevents the influx of neutrophils to the site of carcinogenesis induction [108]. Whether TGF-*β* regulates MMP-8 expression in neutrophils has not been well elucidated yet, although TGF-*β* may decrease MMP-8 expression in human odontoblast [109], and it may be of interest to explore if N2 transition induced by TGF-*β* requires downregulation of MMP-8.

It has recently been described that MMP-8 expression in breast cancer cells provokes a reduction in the microRNA-21 (miR-21), which may predict a recurrence and unfavorable survival in nonsmall cell lung cancer [110]. The miR-21 in turn is induced by TGF-*β* during the EMT [111]. The reduction of miR-21 expression by MMP-8 in turn leads to the inhibition of tumor growth and lung metastasis subsequently with a reduction of TGF-*β* signaling, thus indicating a new way for MMP-8 contribution to decrease tumor progression [112].

### 10.3. Tumor-Associated Dendritic Cells

Dendritic cells (DC) are critical regulators of host immune response that serve as a bridge between innate and adaptive immunity [113]. Dendritic cells play an essential role in the activation of specific immunity since they are the most effective antigen-presenting cells. However, tumor associated dendritic cells (TADCs), which infiltrate neoplastic tissues in response to tumor-derived chemokines, are functionally defective as they have an immature phenotype because of the immunosuppressive cytokines in the tumor microenvironment, such as TGF-*β* and IL-10 [81, 114]. Immature myeloid DCs promote the expansion of Treg cells associated with TGF-*β*-dependent tumor progression [115]. Therefore, TADCs promote T-cell anergy to tumor antigens and Treg activity [70].

Improvements in dendritic cell function may be possible through the inhibition of TGF-*β* (revised in [116]). TGF-*β* regulates the antigen presentation function of differentiated dendritic cells, and exogenous TGF-*β* administration to lipopolysaccharide-stimulated dendritic cells inhibits the expression of MHC class II and costimulatory molecules. Also, TGF-*β* inhibits the IL-1 and TNF-*α*-induced IL-12 production by DCs. This may result in the inhibition of T-cell activation and differentiation of their antitumor activities, indicating that the blocking of TGF-*β* signaling in DCs is a rational therapy to enhance its suppressive role in cancer development [117]. Conversely, it is possible that these tumor-infiltrating dendritic cells could play a role in the immune response to tumors by processing antigenic proteins released by adjacent tumor cells and presenting immunogenic peptides to competent T cells either inside the tumor or after migration into lymphoid organs [118]. Once more, the balance between antitumorigenic and protumorigenic character of cells is dependent on the switch made by the cytokines in the microenvironment.

Dendritic cells produce MMP-1, MMP-2, MMP-3, MMP-9, MMP-12, MMP-14 and MMP-19 [10, 119, 120]. Overproduction of these MMPs by DCs during infection is associated with a decrease in endothelial PECAM-1 and VE-cadherin in vascular endothelium *in vitro* and *in vivo*, which may contribute to the marked increase in vascular permeability. Thus, opening of endothelial barriers by MMP activity may be a mechanism that allows passage of plasma proteins and inflammatory cells into otherwise privileged compartments. However, further research is necessary to investigate whether the behavior of TADCs is similar to that during infection.

### 10.4. Mast Cells

Mast cells are bone marrow-derived tissue-homing leukocytes; these cells seem to be significant for a variety of biological functions, such as tissue repair and regeneration, angiogenesis, inflammation, and cancer [121]. It has been suggested that mast cells can either promote tumor progression or, supported by clinical data, display some tumor suppressive effects [122]. The tumor secretion of stem cell factor functions as a chemoattracting signal for mast cells; then mast cells produce angiogenic factors and MMPs (MMP-2 and MMP-9) to promote tumor vascularization.

In the same way, TGF-*β* is suggested as one of the most potent attractants for mast cells, since TGF-*β*1 has been shown to cause directed migration of cultured mouse mast cells [123], and might indicate the possibility that the increment of TGF-*β* production in the tumor stroma attracted mast cells to the site of tumor. Mast cells produce TGF-*β* which can also act as a pro-angiogenic factor and strongly regulates Tregs within tumors; in turn Tregs inhibit mast cell progenitors and suppress degranulation of mature mast cells in a TGF-*β*-related fashion. Mast cells in turn inhibit expression of IL-10 by Tregs and promote differentiation of proinflammatory Tregs [124, 125], thus suggesting an immune cell loop during the cancer promotion. Intriguingly, TGF-*β* enhances IL-6 expression and secretion in mast cells, which in turn enhances Th17 differentiation [126]. The IL-17 production by mature Th17 may promote tumor growth and provide resistance to antiangiogenic therapy [127–130], which may provide an indirect way of TGF-*β* and mast cells protumorigenic activities.

Mast cells are essential for late stages of tumor expansion, as is demonstrated by the ablation of mast cells which provoked an increment of tumor cells apoptosis and reduced tumor vascularization. Mast cells also were important for the induction of angiogenesis by expressing MMP-9, since MMP-9 deficiency highly impaired the incidence of invasive tumors in skin carcinogenesis [131, 132]. In rats, the injection of mast cells leads to the acceleration of tumor development. Mast cell enhancement of tumor proliferation was demonstrated by the addition of mast cells to the initial tumor inoculum and by pharmacological inhibition of mast cell degranulation [133]. During the degranulation, mast cells released MMP-2 and MMP-9, as well as other proteases, which can activate latent MMP-3 and MMP-9 [134, 135]. The presence of mast cells can provide the changes necessary for the induction of a permissive tumor stroma, and TGF-*β* and MMPs secreted, together with the other myeloid-infiltrating tumor stroma, may collaborate to promote tumor development and cancer cell invasiveness.

### 10.5. Concluding Remarks

It is believed that TGF-*β* and MMPs produced by the epithelial cancer and local stroma cells contribute to the progression and metastatic potential of tumor, acting on the delicate balance between the matrix and the cellular components in the tumor body, on which the success of cancer development depends.

TGF-*β* modifies the expression, secretion, and activation of MMPs, which in a mutual response activate latent TGF-*β* from ECM. In addition, active MMPs increase the bioavailability of cytokines and growth factors that promote tumor cell proliferation, survival, and invasion, as well as tumor angiogenesis to support tumor growth and metastasis. TGF-*β* and MMPs contribute to the recruitment of MMP-producing immune cells into tumor, such as neutrophils, macrophages, and mast cells, and also suppress their anti-tumor activity. In addition, these cells in response to TGF-*β* can produce angiogenic factors (such as VEGF) which contribute to tumor angiogenesis. Finally, locally active TGF-*β* strongly contributes to the immunosuppressive effects of the tumor cells, by suppressing the activity of cytotoxic T cells, dendritic cells, and natural killer cells allowing tumor to escape/evade the immune cell response. Finally both TGF-*β* and MMPs production stimulates carcinoma cell growth, motility, and invasion.

All the cells present in tumor stroma, including inflammatory cells, as well as tumor cells are able to produce TGF-*β*, while types of MMPs secreted are cell type specific. The main loop in the tumor inflammatory environment would be inflammatory cells (M2-TAMs and N2-TANs especially), production of TGF-*β* and MMPs, reciprocal upregulation of TGF-*β* and MMPs, and consequent recruitment of inflammatory cells closing the loop (Figure 2). At the same time, all three members of the loop have been shown to induce cancer growth and invasion by inducing angiogenesis, by degrading ECM, or by cytokine and chemokine production. The fine tuning of the cells' “antitumorigenic” against “protumorigenic” phenotype is regulated in big part by TGF-*β*, subsequently involving a vast number of different cytokines, chemokines, and enzymes, all together defining the final direction of the tumor. Finally, the system in whole as well as its single participants is balancing between the “good” and “bad” influence, acting against or promoting tumor growth and invasion. The details of fine regulation of tumor behavior are complex and yet to be elucidated.

The understanding of the mechanisms by which TGF-*β*, MMPs, and tumor stroma-infiltrating myeloid cells act may open new avenues which will develop more precise therapies for cancer treatment. Several oncotherapies and clinical trials are ongoing for the components of TGF-*β* signaling [1], and more specific inhibitors have been developed to surgically inhibit MMPs as well [136]. Because some MMPs, such MMP-8 and MMP-12, can be beneficial as antitumor targets, a broad MMP targeting is not recommended, as already demonstrated by the unsuccessful use of broad MMP inhibitors in experimental cancer assays [107]. Due to the fact that many studies have demonstrated the correlation between the density of TAMs and poor prognosis, macrophages have also been considered as a target for clinical therapy [84]. The possibility to deplete the macrophages from tumor, by using a combination of zoledronic acid with sorafenib [137] or by inhibiting macrophages infiltration, by drugs, such as thalidomide, linomide, and pentoxifylline [138], may reduce the tumor growth; nonetheless, these analyses need to be translated to human cancer treatment. A novel study has been recently reported, exploiting membrane mannose receptors mainly expressed in M2 TAMs as targets for PEG-sheddable mannose-modified nanoparticles and producing a platform to carry drugs or modulatory anti-tumor agents to M2-TAMs [139].

The regulation of both TGF-*β* and protumorigenic MMPs (e.g., MMP-9) may directly regulate cancer cells growth, angiogenesis, and invasiveness. TGF-*β* blocking may have profound impact on the balance of M1-M2 TAMs and N1-N2 neutrophils and allow dendritic cells maturation. Blocking may also regulate the excessive recruitment of the components of tumor stroma-infiltrating myeloid cells to finally drive/guide the immune myeloid cells against tumors. A combinatory therapy against TGF-*β*, protumorigenic MMPs, and myeloid immune cells may create an antitumor microenvironment more sensitive to traditional chemotherapies of cancer.

Elucidating the complex interplay and roles of TGF-*β* and MMPs in the tumor, including tumor microenvironment, cancer cells, stroma-infiltrating myeloid cells, and ECM interactions, is critical for understanding their participation in the initiation, progression, metastasis, and eventually uncovering potential combinatory therapeutic targets for future treatment of human cancer.

## Figures and Tables

**Table 1:** Classification of matrix metalloproteinases (adapted from [28, 37]).

Subclasses of MMPs	Name
Interstitial collagenases	MMP-1, -8, -13
Gelatinases	MMP-2, -9
Membrane bound MMPs (MT-MMPs)	MMP-14, -15, -16, -17, -24, -25
Stromelysins	MMP-3, -10, -11
Matrilysins	MMP-7, -26
Enamelysins	MMP-20
Elastases	MMP-12
Others	MMP-19, -21, -23a, -27, -28
